# The need for a standard approach to assessing the functionality of rural community water supplies

**DOI:** 10.1007/s10040-017-1711-0

**Published:** 2018-01-23

**Authors:** Helen Bonsor, Alan MacDonald, Vincent Casey, Richard Carter, Paul Wilson

**Affiliations:** 1 British Geological Survey, Lyell Centre, Research Avenue South, Riccarton, Edinburgh, EH14 4AP UK; 2 WaterAid, 47–49 Durham Street, London, SE11 5JD UK; 3 Richard Carter and Associates Ltd, The Oxlip, Ampthill, Bedfordshire, MK45 2EH UK; 4 British Geological Survey, Dundonald House, Upper Newtownards Road, Ballymiscaw, Belfast, BT4 3SB UK

**Keywords:** Water supply functionality, Borehole, Groundwater development, Rural, Sustainable Development Goals (SDGs)

## Abstract

The Sustainable Development Goals have set an agenda for transformational change in water access, aiming for secure household connections globally. Despite this goal, communal groundwater supplies are likely to remain the main source of improved water supplies for many rural areas in Africa and South Asia for decades to come. Understanding the poor functionality of existing communal supplies remains, therefore, a priority. A critical first step is to establish a sector-wide definition of borehole supply functionality and a standard method of its assessment.

## Résumé

Les objectifs du développement durable ont mis en place un programme de changement transformationnel de l’accès à l’eau, visant à assurer des connexions sûres des ménages à l’échelle mondiale. Malgré cet objectif, les approvisionnements en eaux souterraines à l’échelle communale sont susceptibles de demeurer le sujet principal à améliorer pour de nombreuses régions rurales d’Afrique et d’Asie du Sud pour les décennies à venir. La compréhension de la piètre fonctionnalité des approvisionnements existants à l’échelle communale reste donc une priorité. Une première étape cruciale consiste à établir une définition sectorielle de la fonctionnalité de l’approvisionnement par forage et une méthode normalisée de son évaluation.

## Resumen

Los objetivos de desarrollo sostenible han establecido una agenda para un cambio transformacional en el acceso al agua, con la finalidad de establecer conexiones domiciliarias seguras a nivel mundial. A pesar de este objetivo, es probable que los suministros comunales de agua subterránea sigan siendo la principal fuente de mejoras en el abastecimiento de agua para muchas áreas rurales en África y el sur de Asia en las próximas décadas. Entender la funcionalidad deficiente de los suministros comunales existentes sigue siendo, por lo tanto, una prioridad. Un primer paso crítico es establecer una definición sectorial de la funcionalidad del suministro de los pozos y un método estándar para su evaluación.

## 摘要

可持续发展目标为供水转换变化确定了日程,目标就是为全球每家提供安全的供水连接管道。尽管确定了这一目标,但公共供水可能仍然是非洲和南亚许多农村地区未来几十年改善供水的主要水源。因此,了解现有公共供水功能性差的状况依旧是需要优先考虑的问题。关键的第一步是建立部门范围的钻孔供水功能性的定义及其评价的标准方法。

## Resumo

Os Objetivos de Desenvolvimento Sustentável estabeleceram uma agenda para mudanças de paradigmas no acesso à água, visando abastecimento doméstico em escala global. Apesar desse objetivo, os suprimentos comunitários de águas subterrâneas provavelmente continuarão a ser a principal fonte de abastecimento de água potável para muitas áreas rurais da África e do sul da Ásia por décadas. Compreender a baixa funcionalidade desses atuais suprimentos comunitários torna-se, portanto, prioritária. Um primeiro passo crítico é estabelecer uma definição setorial de funcionalidade de fornecimento de poços e um método padrão de avaliação.

## Poor functionality: a stubborn concern in rural water supply

The development of groundwater resources by drilling boreholes and equipping them with handpumps has been fundamental to increased access to safe water across rural Africa and South Asia (MacDonald and Calow 2009; Howard et al. 2016). Between 1990 and 2015, 1.2 billion people in South Asia and sub-Saharan Africa gained access to improved water sources from boreholes, wells and springs, more than halving the number of people reliant on unsafe supplies from rivers and ponds (JMP 2014). However, these positive statistics mask stubbornly high rates of poor functionality and service levels in community boreholes when taking account of quantity, quality, access and reliability. Estimates of the number of non-functional water points vary from 15 to 50% at any one time between different studies (Harvey and Reed 2006; Lockwood and Smits 2011; Banks and Furey 2016). These estimates of functionality have persisted since the 1970s, despite different approaches to introduction of services such as an increased emphasis on demand responsive approaches and community management (Cairncross et al. 1980; Arlosoroff et al. 1987; McPherson and McGarry 1987; Carter and Ross 2016; Whaley and Cleaver 2017). As a result, the original investment and the intended benefits (improved health, nutrition, time-savings and education) are lost for the communities affected (Hunter et al. 2010; UN 2013).

Being able to understand the relative drivers of existing functionality of rural borehole supplies across different settings is essential for future investment and interventions to be able to deliver water supply services of increased sustainability and lasting benefit. However, in the absence of a sector-wide definition of borehole functionality, it is currently difficult to compare existing estimates of functionality accurately (Harvey and Reed 2006; Banks and Furey 2016). There is now a growing research community focussed on this issue as well as efforts to standardise definitions led by the Sanitation and Water For All coalition (Wilson et al. 2016). This essay discusses the implications drawn from a review of this growing literature and suggests guidelines for defining and assessing functionality as a first step to being able to confidently compare studies and understand the relative drivers of poor functionality across different settings.

## Lack of a standard definition

**Fig. 1:**
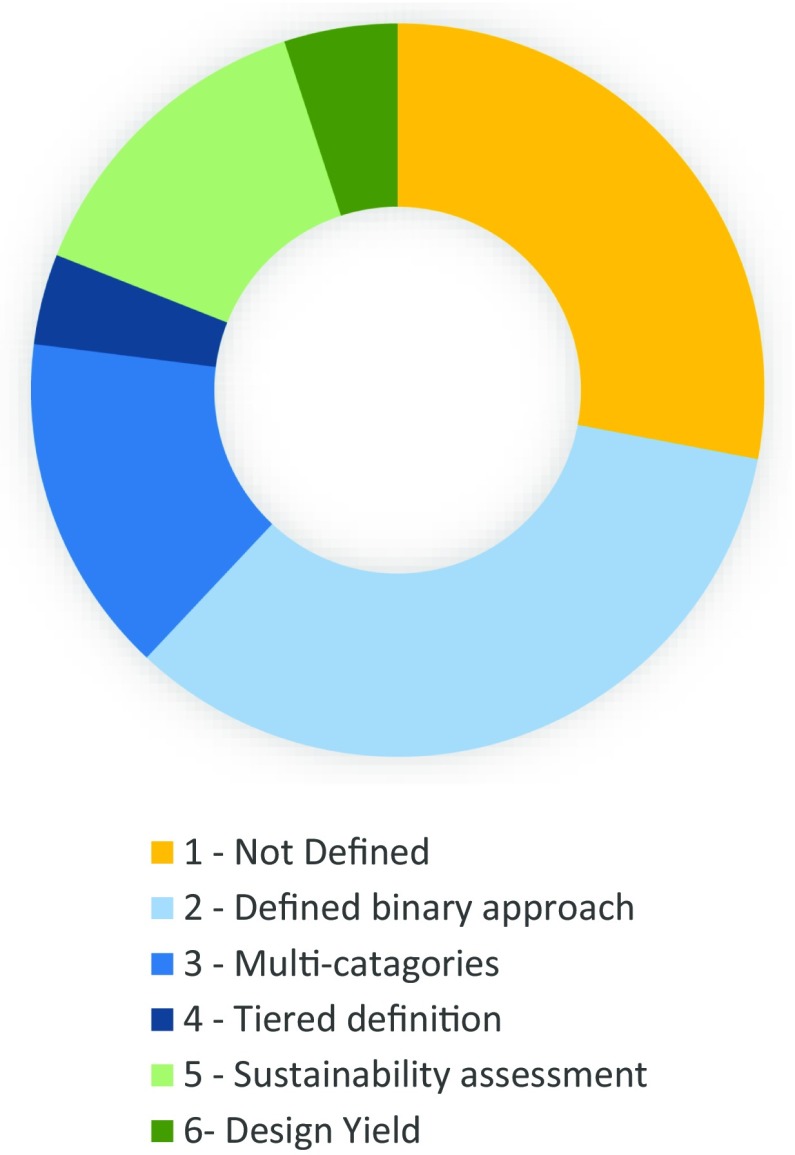
The proportions of published studies employing different approaches to define water point functionality

Currently, there is no single accepted definition of functionality, or what constitutes a functioning water point. This inhibits the ability of the research community, government, donors and practitioners alike to be able to understand the inter-related causes of water point failure. Consequently, solutions may be proposed and implemented that do not address the most significant drivers or deliver lasting benefit. A recent review of 111 studies from published and grey literature (for details see Wilson et al. 2016) found six main categories of how functionality is defined within studies (Table 1; Fig. 1). Within these categories, functionality is mostly measured using qualitative approaches and direct quantitative assessment measurements of functionality are rare.

**Table 1:** The six main approaches used to define and assess water point functionality (details in Wilson et al. 2016)

Definition class	Summary
1. Not defined	Functionality not explicitly defined: by default, working or not working
2. Defined binary approach	Defined to be ‘working’ or ‘not working’ based on whether the water point is working at the time of the visit: ‘in use’/‘not in use’
3. Multi-categories	Different categories are used to capture the different levels of functionality status: functional, minimally functional, functioning through difficulties, broken, missing parts, seasonal
4. Tiered definition	Several different levels of assessment are used to assess functionality. As a minimum, functionality is assessed using a binary approach of ‘working’/‘not working’, but can be examined in greater detail using several levels of assessment
5. Sustainability assessment	A broader assessment approach which includes several factors indicating the reliability of the supply
6. Design yield	A water point is functional if it produces the design yield at the time of the visit

Thirty-four percent of the studies employ a simple binary approach to define water point functionality based on whether the source is ‘in use’ or ‘not in use’ at the time of the visit. A further 28% of the studies reviewed were found not to explicitly define functionality, but by default usually assume a simple binary working/not working definition. The limitation of a simple binary approach has led some to define multiple categories such as *partially functioning* but this in itself has made comparison of surveys more difficult. A tiered approach has been advocated by a few (4%) recent studies (Leclert 2012; Tincani et al. 2015; Carter and Ross 2016) in which several different levels of assessment and indicators are used to assess functionality. Fourteen percent consider functionality as a category within a much broader sustainability assessment of water service, but definitions used within this are often unclear.

Three major projects (Triple 2009; Cross et al. 2013; Prat et al. 2015) have highlighted the need for clearer definitions of water point functionality in order to be able to understand, and move towards, improving service sustainability. These projects and related literature have drawn attention to the relationship between functionality and sustainability (Duti 2012; Moriarty et al. 2013; Prat et al. 2015). Indeed, Lockwood and Smits (2011) observe that functionality (which they express as the percentage of water points working at any given time) often serves as a proxy for sustainability; however, it is important to recognise that the two are not synonymous, as noted by Carter and Ross (2016). Water points can be non-functional at the moment they are inspected but, with an effective maintenance system may, over the course of a year, have limited downtime and deliver a sustainable supply of water. Equally, other water points which are found to be functioning at the time of inspection, may in reality experience significant downtime over a year, due to fundamental faults or issues, or less effective maintenance, and are less likely to deliver a sustainable supply (Carter and Ross 2016).

## Guidelines for assessing functionality

A major drawback of having such different definitions of functionality, and more worrying, no definition, is that it is difficult to compare studies. The term “functionality” is used by studies to represent both functionality at a point in time (‘snapshot’) and the performance of a water point, which includes a temporal/ reliability dimension. It is essential to differentiate between the two, and understand to which domain different functionality figures relate. Taking the findings from the literature review and developing thinking from an earlier paper (Carter and Ross 2016), it is suggested here that the following guidelines should form core criteria for assessing functionality of water points:

- Functionality should be measured against an explicitly stated standard and population of water points.
- Functionality should be measured separately from the users’ experience of the service it provides.
- The assessments should be tiered, allowing for further information, but always being able to be reduced to a simple measure.
- A distinction can be made between surveying functionality as a snapshot (e.g. for national metrics) and monitoring individual water point performance and reliability (including a temporal aspect).

These guidelines are applied in the following to develop a tiered approach for defining functionality of boreholes equipped with handpumps. The tiered approach enables functionality to be assessed in terms of the performance of that water point with an increasing level of detail:

1. Binary: Is the water point physically working and producing some water at the time of the survey visit? (yes/no)
2. Yield snapshot: Does the water point provide the sufficient minimum design yield (for example, 10 L/min)?
3. Reliable yield: Does the water point provide a reliable yield (meeting criteria 1 and 2 above) year round (less than 30 days downtime in the previous 12 months)?
4. Reliable yield including water quality: Does the water supply pass WHO inorganic and pathogen guidelines, as well as provide a reliable yield year-round?

At the highest level, this approach assesses functionality based on a binary definition of ‘working’/ ‘not working’ at the time of a survey. The subsequent levels of assessment beneath this binary definition then move to provide a more detailed understanding of the yield and reliability of supply. This enables a more refined assessment of functionality to be undertaken where possible, whilst acknowledging that such detailed assessments are not feasible or appropriate in all cases. The ‘binary’ and ‘yield snapshot’ assessments match the requirements of national survey assessments, whilst the more performance-focused definitions, which assess the reliability of the functionality, are more relevant to local or regional surveys looking to track the functionality of individual water points or programmes through time.

## Measuring functionality performance

The set of aforementioned tiered assessment approaches require different quantities of data to be collected, and therefore increasing resources of time and cost. Importantly, the assessment criteria for the binary functionality definition at the highest level of the tiered approach is accessible to all survey types, and ensures a minimum level of functionality data can be compared between studies.

**Fig. 2:**
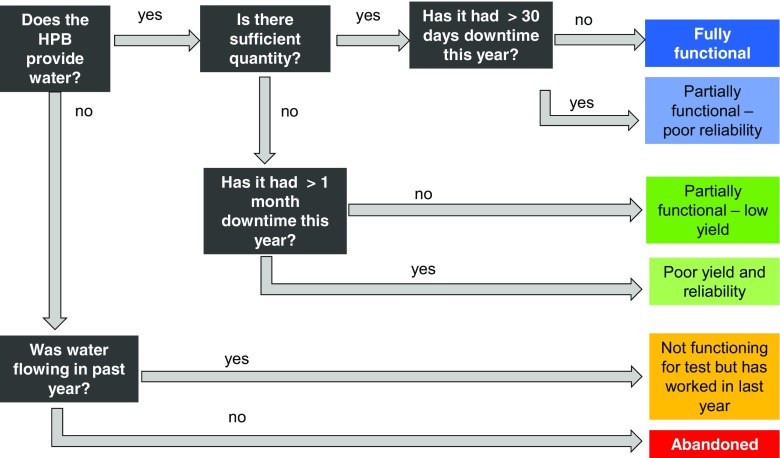
An approach to assess the functionality of a water point according to the reliability of yield. This performance assessment includes a temporal dimension of the water point’s reliability

The tiered approach also enables a clear distinction to be made between data collated to assess functionality at a point in time (tiers 1 and 2), and the data required to assess reliability of the water point functionality over time – i.e. reliability (tier 3). Figure 2 shows an assessment approach and the different levels of information which must be collated to assess the functionality of a water point according to different tiers in the approach. A tier 1 assessment could be undertaken by just answering the first question (Fig. 2), or a tier 2 assessment by answering the first two questions. All the questions need to be answered to complete a tier 3 assessment.

Good statistical design can be used to gain maximum information for limited resources. A tier 1 assessment can be undertaken rapidly for an entire domain—for example a district, region, or even country. Good sampling design could then enable a smaller sample to be assessed within the domain at tiers 2, 3 or 4, and the result used to estimate results across the entire domain.

## More than a definition: creating the necessary framework to understanding the underlying causes of community water supply failure

Defining and measuring functionality is only a starting point. Water point failure and functionality is a multi-faceted, multi-layered issue, with growing complexity emerging as one looks beyond the immediate causes of failure. If the SDGs are to be met, and water supply service across the world to become increasing sustainable and offer acceptable service levels (UNDP 2016), the relative reasons for poor functionality in different environments and cultures need to be understood. Earlier work by the authors (Bonsor et al. 2015) identified a pathway for examining causes of poor functionality that includes: *primary causes* (e.g. mechanical failure, reduced yield, poor water quality); *secondary causes* (e.g. geology, poor siting, lack of spare parts, basic maintenance, local governance arrangements); the *underlying conditions* (including the institutional, financial and social factors that shape an environment in which failure is more or less likely); and *long-term trends* (e.g. changes in demand for water, evolution of governance arrangements, reduction in regional groundwater availability, climate change, deterioration of water quality). Given the scale of the problem, many are now working to understand poor functionality, and there are increasing efforts to standardise definitions and existing indicators. This provides an opportunity to reflect and consolidate approaches. By improving definitions, and the systematic collection of data pertaining to failure, efforts in the sector could be further focussed to better understand the primary drivers of functionality in different settings, and the specific interventions which will be able to deliver lasting benefit.
